# TCLNet: A Thermal Contrast-Aware Lightweight Network for infrared object detection

**DOI:** 10.1371/journal.pone.0361114

**Published:** 2026-10-22

**Authors:** Xiyuan Zhu, Hongtao Dang, Xiaoyuan Jin

**Affiliations:** Xijing University, Xi’an, Shaanxi, China; University of Electronic Science and Technology of China, CHINA

## Abstract

Infrared object detection plays an important role in low-light and complex-environment perception. However, infrared images often exhibit low signal-to-noise ratio, weak target-background contrast, and limited texture information, which can degrade detection performance in small-target and cluttered-background scenarios while making it difficult to balance detection accuracy and model complexity. To address these challenges, this paper proposes TCLNet, a Thermal Contrast-Aware Lightweight Network for infrared object detection. TCLNet introduces a Thermal Contrast Encoder (TCE) to enhance local contrast-sensitive target representations and a Thermal-Guided Cross-Feature Attention (TGCA) mechanism to promote interactions between contrast-enhanced and semantic features, thereby improving target discrimination and suppressing background interference.Experiments were conducted independently on the M3FD, FLIR ADAS v2 (denoted as FLIR_2), and NUDT-SIRST1 datasets. TCLNet achieves mAP50 values of 60.5%, 45.4%, and 91.4%, respectively, corresponding to improvements of 12.8, 11.3, and 7.5 percentage points over the YOLO12n baseline. Meanwhile, the model maintains a compact size of approximately 2M parameters. These results demonstrate that TCLNet improves infrared target detection, particularly in low-SNR and small-target conditions, while achieving a favorable balance between detection accuracy and model complexity.

## 1. Introduction

Infrared object detection is important for nighttime monitoring, autonomous driving, industrial inspection, and intelligent transportation. Unlike visible-light cameras, infrared sensors record scene-dependent intensity patterns influenced by scene radiance, object properties, and sensor response. Infrared imaging can therefore provide complementary visual information under low illumination and certain adverse environmental conditions [1,2].

Despite these advantages, infrared images present several challenges for object detection. First, targets often exhibit weak contrast relative to complex backgrounds because of low signal-to-noise ratio (SNR) or low signal-to-clutter ratio (SCR). Second, limited spatial resolution, sensor noise, and smooth intensity transitions can produce indistinct target boundaries. Third, the absence of chromatic information and the scarcity of detailed textures restrict the discriminative capacity of conventional feature representations. These limitations are particularly severe for small targets, which may occupy only a few pixels and can be progressively weakened during feature downsampling [3].

Infrared intensity patterns are related to scene radiance but should not be directly interpreted as calibrated temperature measurements. Their values are also influenced by sensor characteristics, object emissivity, environmental conditions, and image preprocessing [4]. Nevertheless, infrared targets frequently exhibit local intensity differences and spatial response variations relative to their surrounding backgrounds. These observable patterns motivate the use of relative-contrast and gradient-based feature cues. Such cues provide a thermally inspired inductive bias without requiring explicit recovery of temperature, radiance, or heat-diffusion parameters [5,6].

Recent advances in deep learning have substantially improved general object detection. Existing approaches include two-stage convolutional detectors, such as the R-CNN family [7–12], one-stage detectors, such as the YOLO family [13–17], and transformer-based detectors represented by DETR and its variants [18–21]. Although these detectors perform well on visible-light benchmarks, their architectures are not specifically designed for the weak contrast and limited texture commonly observed in infrared images. Directly applying them to infrared data may produce suboptimal feature representations, particularly for small targets and cluttered backgrounds.

To address these limitations, this paper proposes TCLNet, a Thermal Contrast-Aware Lightweight Network for infrared object detection. TCLNet is constructed using a lightweight YOLO-based framework and introduces contrast-sensitive feature enhancement and cross-feature attention. Instead of estimating physical thermal quantities, the proposed network learns relative target-background responses from infrared features. It then uses these responses to guide the interaction between contrast-enhanced and semantic representations. This design is intended to improve target discrimination while retaining a compact parameter count.

The main contributions of this study are summarized as follows:

1. Thermal Contrast Encoder (TCE): A thermally inspired feature-enhancement module is introduced to characterize local target-background contrast and spatial response variations. TCE combines learned response projection, local background-response estimation, normalized relative-contrast calculation, and Sobel-gradient extraction. These operations are used to generate a contrast-sensitive modulation map for infrared feature enhancement.
2. Thermal-Guided Cross-Feature Attention (TGCA): A local cross-attention mechanism is developed to facilitate interaction between contrast-enhanced and semantic features. The contrast-enhanced features generate the Query, while the original semantic features generate the Key and Value. A local-window strategy reduces the computational cost of global attention and limits interactions with spatially distant background regions.
3. Thermal-Attentive Fusion C2f (TAF-C2f): TCE and TGCA are integrated into a lightweight feature-extraction block. The resulting module jointly optimizes contrast-sensitive enhancement, local cross-attention, and semantic feature extraction within a compact parameter budget.

The remainder of this paper is organized as follows. Section 2 reviews related work on infrared object detection. Section 3 describes the TCLNet architecture and its main components. Section 4 presents the datasets, evaluation metrics, and implementation details. Section 5 reports the comparative experiments, ablation studies, visual analysis, and robustness evaluation. Section 6 concludes the paper and discusses possible directions for future research.

## 2. Related work

### 2.1. Traditional Infrared Target Detection Methods

The early infrared target detection methods mainly relied on manual feature design and signal processing techniques, such as the detection method based on Haar features and AdaBoost cascade classifier [22], the HOG feature method [23], and the detection strategy based on local feature transformation [24]. These methods usually construct specific feature descriptors to distinguish targets from backgrounds, achieving certain results in simple scenarios. However, due to the common characteristics of infrared images, such as low contrast, weak texture, and low signal-to-noise ratio, traditional artificial features often have difficulty accurately depicting the complex feature distribution of infrared targets. Moreover, these methods are sensitive to environmental changes and complex backgrounds, and are prone to false detections or missed detections in strong noise or cluttered environments, thus having significant limitations in robustness and generalization in complex infrared scenarios.

To improve the performance of infrared target detection, some studies have introduced statistical modeling and signal processing methods to separate targets from backgrounds. For example, Gao et al. [25] proposed an infrared patch image model, achieving target-background separation through low-rank sparse decomposition, but this method has high computational complexity and is difficult to meet real-time requirements in practical applications. Chen et al. [26] proposed a local contrast detection method based on the human visual system (HVS), which to some extent alleviated the missed detection problem of low-signal-to-noise ratio targets, but still had a relatively high false alarm rate in complex cluttered backgrounds. Guo et al. [27] proposed a frequency-domain saliency detection method based on phase spectrum, improving detection efficiency through frequency-domain analysis, but single-scale analysis is prone to losing target detail information. Wang et al. [28] used robust principal component analysis (RPCA) for infrared patch decomposition, improving detection robustness in strong noise environments, but the computational cost is relatively high. Guo and Zhang [29] proposed a detection model based on quaternion saliency, promoting the development of multimodal detection, but their method relies on multimodal input data and has a more complex calculation process.

### 2.2. Deep learning-based infrared target detection

With the development of deep learning technology, infrared target detection methods based on convolutional neural networks have gradually become a research hotspot. Deep networks can automatically learn multi-level feature representations through end-to-end training, demonstrating stronger target expression capabilities in complex backgrounds. Dai et al. [30] proposed a single-stage cascaded refinement network, enhancing the accuracy of infrared small target detection through a multi-stage feature refinement mechanism. Yao et al. [31] combined traditional filtering methods with convolutional neural networks to enhance the network’s response ability to weak targets. Du et al. [32] proposed an infrared target detection framework based on temporal features, strengthening the features of moving targets through the inter-frame energy accumulation mechanism, and improving the detection accuracy by combining an improved intersection-over-union strategy. Dai et al. [33] further proposed an asymmetric context modulation method (ACM), enhancing the target feature expression ability by introducing context information, thereby improving the detection performance in complex scenarios.

Although deep learning methods have achieved significant progress in the field of infrared target detection, most existing methods directly adopt the visible light detection framework and local target-background intensity differences and spatial response variations. Therefore, in low signal-to-noise ratio, small targets, and complex background scenarios, there is still considerable room for improvement in detection performance.

### 2.3. Lightweight infrared object detection networks

In practical applications, infrared target detection systems are typically deployed on embedded devices or edge computing platforms, making the computational complexity and real-time performance of the model equally crucial. In recent years, research on lightweight target detection networks in infrared scenarios has gradually increased. Fan et al. [34] proposed the LUD-YOLO lightweight detection network, which enhances multi-scale target detection capabilities by introducing a progressive feature pyramid structure. However, there is still a category confusion problem in dense scenarios. Li et al. [35] designed the EGC lightweight convolution module and constructed the EGC-CSPGhostNet backbone network, reducing the model’s parameter quantity while maintaining good detection performance. However, their mAP metric still has a certain degree of decline. Chen et al. [36] proposed the TCI-Former model, which builds a Transformer structure based on the heat conduction thermodynamics principle to enhance infrared feature expression capabilities. However, the parameter scale and FLOPs of this method are relatively high, and its deployment on resource-constrained platforms still faces certain challenges.

In addition to infrared detection, lightweight YOLO-based detectors have also been applied to other practical industrial and agricultural scenarios. Nakjai et al. [37] proposed an object detection approach to egg tray corner counting based on YOLOv12. By treating egg-tray corners as detection targets, their method transformed the counting task into a real-time object detection problem, demonstrating the applicability of YOLO-based frameworks in domain-specific counting and quality inspection scenarios. However, such methods mainly focus on visible-light structured objects and do not specifically address the low contrast, weak texture, and thermal diffusion characteristics of infrared images.

In summary, the existing infrared target detection methods still have certain limitations in feature expression capabilities or computational efficiency. How to maintain the model’s lightweight while fully utilizing the thermal physical features in infrared images and improving detection performance in complex scenarios remains an important research direction at present. Therefore, this paper proposes a Thermal Contrast-Aware Lightweight Network for Infrared Object Detection (TCLNet), which effectively improves the detection accuracy and efficiency of infrared targets by explicitly modeling the thermal contrast information in infrared images and combining a lightweight attention mechanism.

## 3. Method

### 3.1. Thermal Contrast Encoder Module (TCE)

Infrared targets frequently exhibit local intensity differences relative to their surrounding backgrounds. However, the pixel values of an infrared image are influenced by sensor response, imaging conditions, emissivity, environmental factors, and image normalization. Therefore, an uncalibrated infrared image cannot be directly interpreted as a quantitative measurement of physical temperature or thermal radiation. Based on this consideration, the Thermal Contrast Encoder (TCE) is designed as a thermally inspired feature-learning module. It aims to learn local target-background contrast and spatial variation cues that are useful for infrared target detection.

As illustrated in Fig 1, the TCE consists of three main operations: learned response projection, local background-response estimation, and contrast-gradient feature modulation. Given an input feature map $X\in R^{B\times C\times H\times W}$, TCE first projects the multi-channel feature map into a normalized single-channel response map:

**Fig 1:**
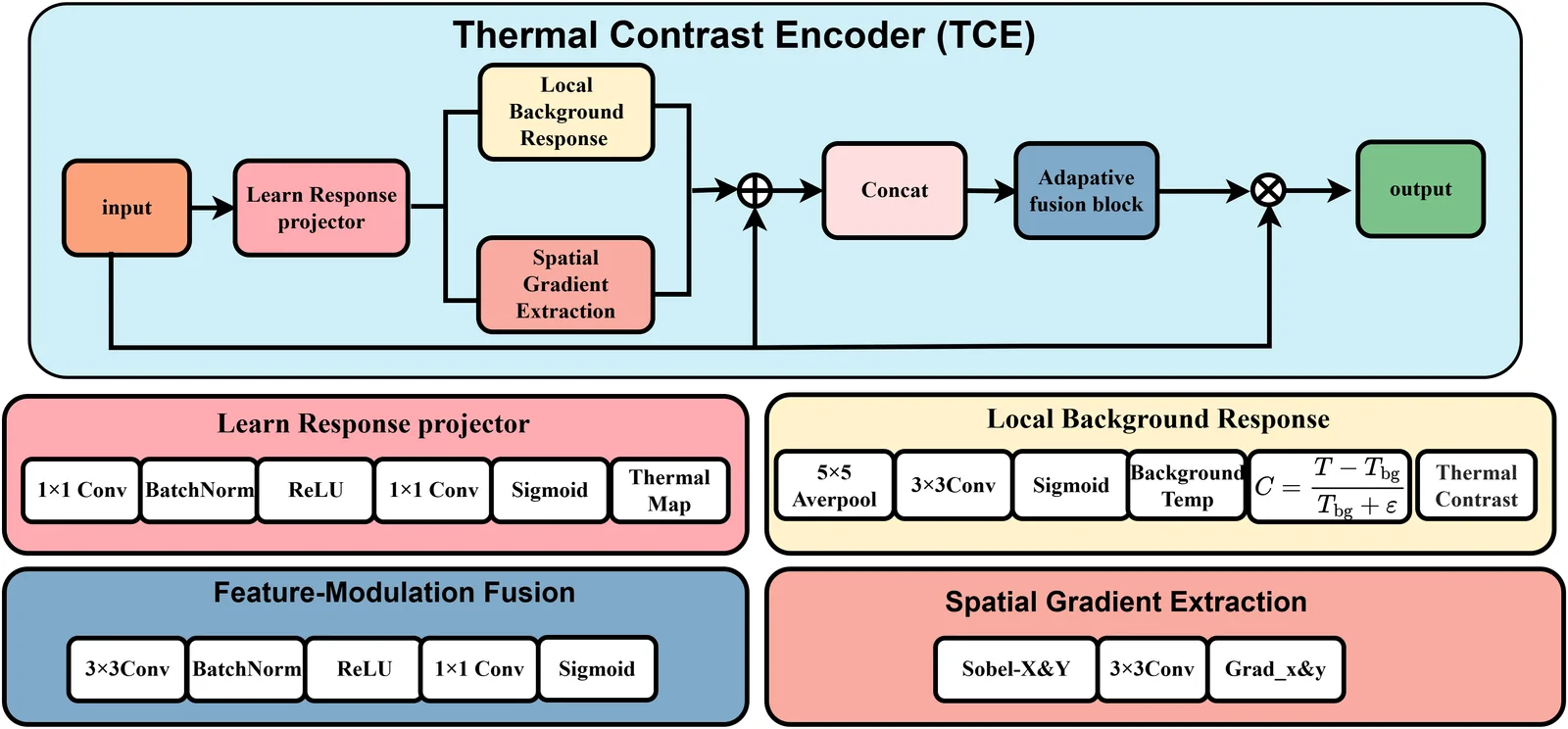
Structure of the Thermal Contrast Encoder (TCE).


$$\begin{array}{c} \text{R}=\sigma \left({\text{f}}_{\text{2}}\left(\delta \left(\text{BN}\left({\text{f}}_{\text{1}}\left(\text{X}\right)\right)\right)\right)\right) \end{array}$$ (1)

where $f_{\text{1}}$ and $f_{\text{2}}$ denote two successive 1×1 convolutional layers, BN(·) represents batch normalization, δ(·) denotes the ReLU activation function, and σ(·) is the Sigmoid function. The resulting map $R\in [\text{0},\text{1}{]}^{B\times \text{1}\times H\times W}\;$represents a learned response that emphasizes infrared-relevant intensity patterns.

To obtain a local reference for target-background comparison, the projected response is processed using 5×5 average pooling followed by a 3×3 convolution:


$$\begin{array}{c} {\text{R}}_{\text{b}}=\sigma \left(\text{BN}\left({\text{g}}_{\text{3}\times \text{3}}\left({\text{AvgPool}}_{\text{5}\times \text{5}}(\text{R})\right)\right)\right) \end{array}$$ (2)

where $g_{\text{3}\times \text{3}}(\cdot )$ denotes a 3×3 convolution and $R_{b}\;$represents the estimated local background response. Average pooling suppresses rapid local variations and provides a smoothed contextual reference.

Based on the projected response and its local background reference, a normalized relative-contrast map is calculated as:


$$\begin{array}{c} C_{r}=clip\left(\frac{R-R_{b}}{R_{b}+\epsilon },-\text{1},\text{1}\right) \end{array}$$ (3)

where $\varepsilon =\text{1}{\text{0}}^{-\text{6}}$ is introduced to avoid numerical instability. The clipping operation limits excessively large responses and stabilizes feature optimization. The resulting $C_{r}$ describes the relative deviation of the learned response from its local context, thereby providing a contrast-sensitive representation under varying infrared backgrounds.

Local contrast alone does not fully describe the spatial structure of small infrared targets. Therefore, TCE additionally extracts spatial response variations using fixed Sobel operators. The horizontal and vertical Sobel kernels are defined as:


$$\begin{array}{c} {\text{K}}_{\text{x}}=[\begin{array}{ccc} -\text{1} & \text{0} & \text{1}\\ -\text{2} & \text{0} & \text{2}\\ -\text{1} & \text{0} & \text{1} \end{array}],\;{\text{K}}_{\text{y}}=[\begin{array}{ccc} -\text{1} & -\text{2} & -\text{1}\\ \text{0} & \text{0} & \text{0}\\ \text{1} & \text{2} & \text{1} \end{array}] \end{array}$$ (4)

The horizontal and vertical response gradients are then calculated by:


$$\begin{array}{c} {\text{G}}_{\text{x}}={\text{K}}_{\text{x}}*\text{R},\;\;{\text{G}}_{\text{y}}={\text{K}}_{\text{y}}*\text{R},\;\;\text{G}={\text{G}}_{\text{x}}+{\text{G}}_{\text{y}} \end{array}$$ (5)

where * denotes the two-dimensional convolution operation. The aggregated gradient response G provides complementary information about local boundaries and spatial intensity variations.

Finally, the original feature map X, relative-contrast map $C_{r}$, and aggregated gradient response G are concatenated along the channel dimension. A feature-modulation map is generated through a fusion function:


$$\begin{array}{c} A=\sigma \left(f_{\text{1}\times \text{1}}\left(\delta \left(\text{BN}\left(f_{\text{3}\times \text{3}}\left([X,C_{r},G]\right)\right)\right)\right)\right),\;\;Y=X⊙A \end{array}$$ (6)

where [·] denotes channel-wise concatenation, ⊙ represents element-wise multiplication, and ϕ(·) consists of a 3×3 convolution, batch normalization, a SiLU activation function, and a 1×1convolution. The generated modulation map $A\in [\text{0},\text{1}{]}^{B\times C\times H\times W}$ adaptively adjusts the original features, producing the enhanced output Y.

Through these operations, TCE introduces a thermally inspired inductive bias by jointly considering local relative contrast and spatial response variations. The module is designed to improve the sensitivity of the detector to compact target-related intensity patterns while reducing responses to locally consistent backgrounds. However, TCE does not recover absolute temperature, calibrated radiance, emissivity, or physical heat-diffusion parameters. Its outputs should therefore be interpreted as learned infrared contrast-sensitive features, and its effectiveness is evaluated through the detection and ablation experiments presented in the following sections.

### 3.2. Thermal-Guided Cross-Feature Attention Module (TGCA)

Infrared target features may be weakened by downsampling or dominated by complex background responses during deep feature extraction. To improve the interaction between contrast-sensitive and semantic representations, we propose a Thermal-Guided Cross-Feature Attention module (TGCA), as illustrated in Fig 2. The term “cross-feature” indicates that the attention interaction is performed between two feature branches, namely the contrast-enhanced branch and the semantic branch.

**Fig 2:**
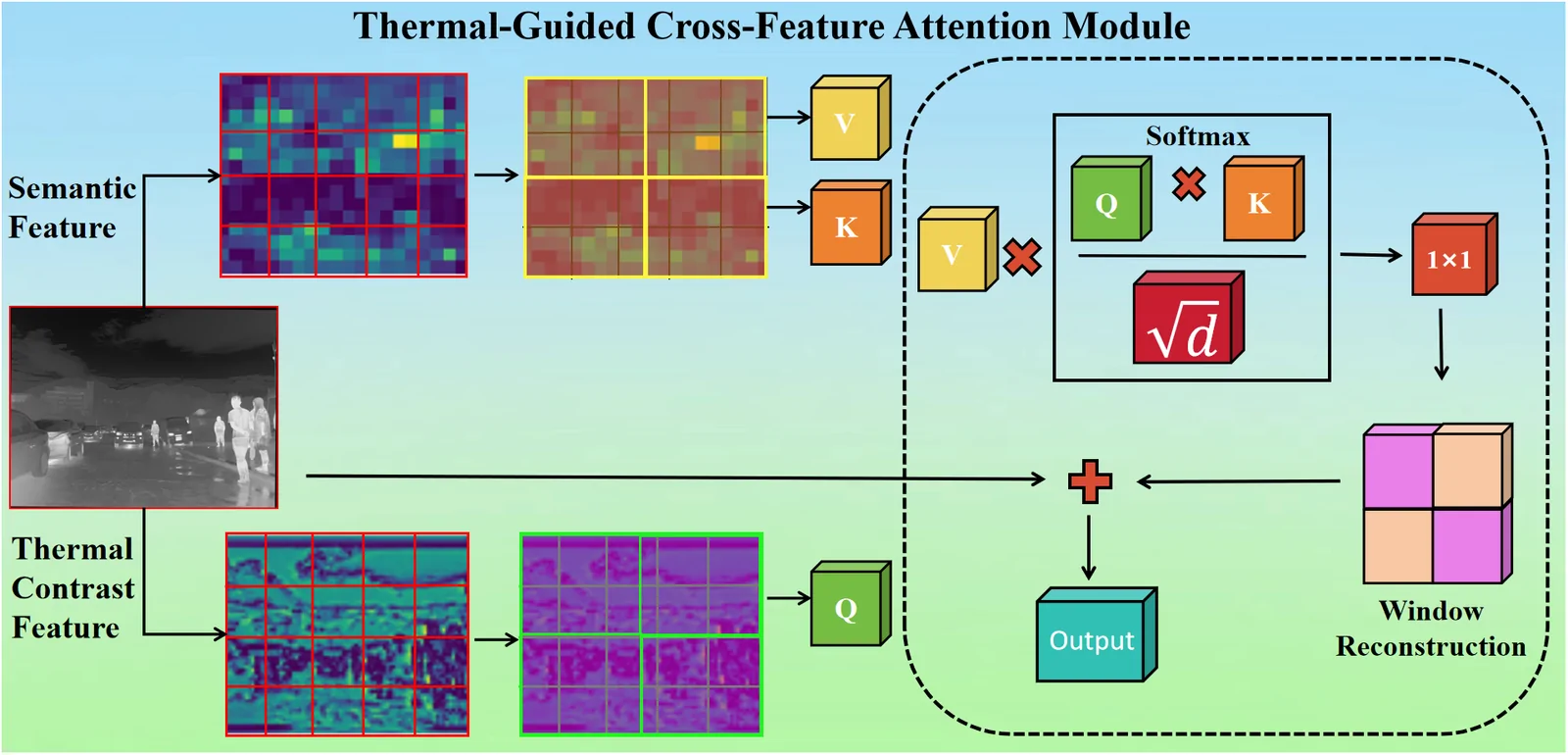
Structure of the Thermal-Guided Cross-Feature Attention module.

Given a semantic feature map$\;X\in R^{B\times C\times H\times W}$, the TCE module produces a contrast-enhanced feature map $X_{c}\in R^{B\times C\times H\times W}$ with the same dimensions. Unlike conventional self-attention, TGCA generates the Query from $X_{c}$, while the Key and Value are generated from X. The corresponding projections are implemented using lightweight 1×1 convolutions:


$$\begin{array}{c} \text{Q}={\text{P}}_{\text{q}}({\text{X}}_{\text{c}}),[\text{K},\text{V}]={\text{P}}_{\text{kv}}\left(\text{X}\right) \end{array}$$ (7)

where $P_{q}(\cdot )$ and $P_{kv}(\cdot )$ denote the Query and Key-Value projection functions, respectively. This branch-asymmetric design enables the contrast-sensitive responses extracted by TCE to guide the interaction among semantic features.

Global attention over N=H×W spatial tokens has a computational complexity of $O(N^{\text{2}}d)$, which becomes computationally expensive for high-resolution feature maps. To reduce this cost, TGCA divides the input feature maps into non-overlapping local windows. Let WindowPartition(·) divide X and$\;{\text{X}}_{\text{c}}$ into L local windows, where each window contains M×M tokens. In this study, the window size is set to M = 12:


$$\begin{array}{c} X_{w}{w=\text{1}}^{L}=\text{WindowPartition}(X),\;\;{Xc,w}_{w=\text{1}}^{L}=\text{WindowPartition}(X_{c}) \end{array}$$ (8)

When the spatial dimensions of the feature map are not divisible by the predefined window size, bilinear interpolation is employed for spatial size adaptation before window partitioning. After the attention operation, the resulting feature map is restored to its original spatial resolution. With this local-window strategy, the computational complexity of attention is reduced from global $O(N^{\text{2}}d)$ to approximately $O(LM^{\text{4}}d)$, since each window contains $M^{\text{2}}$ tokens.

For each local window w and attention head j, the projected features are denoted as $Q_{w}^{\left(j\right)},K_{w}^{\left(j\right)},and{\;V}_{w}^{\left(j\right)},$ respectively, where $\;{\text{Q}}_{\text{w}}^{\left(\text{j}\right)},{\text{K}}_{\text{w}}^{\left(\text{j}\right)},{\text{V}}_{\text{w}}^{\left(\text{j}\right)}$. The attention weights are calculated using scaled dot-product attention:


$$\begin{array}{c} A_{w}^{(j)}=Dropout\left[Softmax\left(\frac{Q_{w}^{(j)}{\left(K_{w}^{(j)}\right)}^{\text{T}}}{\sqrt{d}}\right)\right] \end{array}$$ (9)

where d denotes the feature dimension of each attention head. In our implementation, the number of attention heads is set to $N_{h}=\text{2}$, and the dropout probability is set to 0.1. A larger value in $A_{w}^{\left(j\right)}$ represents a stronger affinity between a contrast-enhanced query token and its corresponding semantic key tokens within the same local window.

The attended feature for each local window is subsequently obtained as:


$$\begin{array}{c} {\text{O}}_{\text{w}}=P_{o}\left[{\text{Concat}}_{j=\text{1}}^{N_{h}}\left(A_{w}^{\left(j\right)}V_{w}^{\left(j\right)}\right)\right] \end{array}$$ (10)

where $P_{o}(\cdot )$ denotes a 1×1 convolutional output projection. The outputs from all local windows are then rearranged to reconstruct the complete spatial feature map:


$$\begin{array}{c} \text{O}=\text{BN}\left[\text{WindowReverse}\left({O_{w}}_{w=\text{1}}^{L}\right)\right] \end{array}$$ (11)

Finally, a residual connection is introduced to preserve the original semantic information and stabilize feature optimization:


$$\begin{array}{c} Y=X+O \end{array}$$ (12)

where $Y\in R^{B\times C\times H\times W}$ denotes the final output of TGCA. Through this design, TGCA exploits the contrast-sensitive representations generated by TCE to guide local semantic feature interactions, while avoiding the high computational cost associated with global attention. It should be noted that the proposed module operates entirely on learned feature representations and does not explicitly estimate physical temperature or model heat-diffusion processes.

### 3.3. Thermal-Attentive Fusion C2f Module (TAF-C2f)

The conventional C2f module provides efficient feature extraction through its multi-branch structure and partial feature reuse mechanism. However, its generic feature transformation strategy is not specifically designed for infrared imagery, where targets are often characterized by weak contrast, limited texture, and strong background interference. Directly stacking additional enhancement or attention modules may improve feature representation but can also introduce considerable computational overhead, which is undesirable for lightweight infrared object detection.

To address these limitations, we propose the Thermal-Attentive Fusion C2f (TAF-C2f) module, which integrates the Thermal Contrast Encoder (TCE) and Thermal-Guided Cross-Feature Attention (TGCA) into a lightweight C2f-style feature extraction framework. The proposed design enhances contrast-sensitive target representations and promotes thermal-semantic feature interaction while retaining the efficient feature reuse mechanism.

Given an input feature map$X\in R^{B\times C_{\text{in}}\times H\times W}$, a preprocessing convolution $F_{\text{pre}}(\cdot )$ is first employed to adjust the channel dimension. The resulting feature map is split into two branches along the channel dimension:


$$\begin{array}{c} \left[{\text{Y}}_{\text{0}},{\text{Y}}_{\text{1}}]=\text{Split}({\text{F}}_{\text{pre}}(\text{X})\right) \end{array}$$ (13)

The branch features are then processed by lightweight bottleneck units:.


$$\begin{array}{c} Y_{i+\text{2}}=B_{i}(Y_{i}),\;i=\text{0},\ldots ,n-\text{1}, \end{array}$$ (14)

where $B_{i}(\cdot )$ denotes the i−th bottleneck unit and n is the number of bottleneck units in the block. This operation produces a set of generated branch features $\left\{Y_{\text{2}},\ldots ,Y_{n+\text{1}}\right\}$. The last generated feature is selected as the enhancement branch:


$$\begin{array}{c} X_{t}=Y_{n+\text{1}}. \end{array}$$ (15)

The selected feature is first processed by TCE to generate a contrast-enhanced feature:


$$\begin{array}{c} X_{\text{th}}=\text{TCE}(X_{t}), \end{array}$$ (16)

The TGCA module then models local cross-feature interactions between the semantic feature $X_{t}$ and the contrast-enhanced feature $X_{\text{th}}$. As defined in Section 3.2, TGCA internally applies residual fusion and produces:


$$\begin{array}{c} X_{\text{th}}G_{t}=\text{TGCA}(X_{t},X_{\text{th}})=X_{t}+O_{t}=\text{TCE}(X_{t}), \end{array}$$ (17)

where $O_{t}$ denotes the local cross-feature attention output.

A learnable scaling coefficient α is used to adaptively control the contribution of the TGCA-enhanced feature:


$$\begin{array}{c} {\hat{Y}}_{n+\text{1}}=Y_{n+\text{1}}+\sigma (\alpha )G_{t}. \end{array}$$ (18)

Finally, the generated branch features are concatenated and projected to the output channel dimension:


$$\begin{array}{c} O=F_{\text{out}}\left(F_{\text{3}\times \text{3}}\left(\text{Concat}(Y_{\text{2}},\ldots ,{\hat{Y}}_{n+\text{1}})\right)\right) \end{array}$$ (19)

Here, $F_{\text{3}\times \text{3}}(\cdot )$ denotes the 3×3 fusion convolution and $F_{\text{out}}(\cdot )$ denotes the final 1×1 output projection.

Through this design, TAF-C2f retains the efficient feature reuse strategy of C2f while introducing contrast-sensitive feature modulation and local cross-feature attention. Rather than explicitly estimating physical temperature or heat-diffusion parameters, the module operates on learned infrared feature representations and uses contrast-sensitive cues to guide semantic feature extraction. This helps preserve weak target responses and suppress background interference under low signal-to-noise ratio conditions without introducing excessive computational complexity.

### 3.4. Thermal Contrast-Aware Lightweight Network (TCLNet)

To address weak target responses, low signal-to-noise ratios, and complex background interference in infrared object detection, we propose a Thermal Contrast-Aware Lightweight Network (TCLNet) based on the lightweight YOLO12n framework. The overall architecture is shown in Fig 3, and the detailed network configuration is summarized in Table 1.

**Table 1:** Overview of the TCLNet framework.

Algorithm 1. TCLNet Framework Overview
Input: infrared image I; model parameters Θ
Output: detection results D
1. Resize I to 320×320.
2. Extract shallow feature $F_{\text{0}}=\text{Stem}(I)$.
3. **for each selected TAF-C2f block do**:
4. Project and split feature: $\left[Y_{\text{0}},Y_{\text{1}}]=\text{Split}(F_{\text{pre}}(X)\right)$.
5. Generate branch features: $Y_{i+\text{2}}=B_{i}(Y_{i}),i=\text{0},\ldots ,n-\text{1}$.
6. Select enhancement branch: $X_{t}=Y_{n+\text{1}}$.
7. Compute learned response map: $R=\sigma (f_{\text{2}}(\delta (\text{BN}(f_{\text{1}}(X_{t})))))$.
8. Estimate local background response: $R_{b}=\sigma (\text{BN}(g_{\text{3}\times \text{3}}({\text{AvgPool}}_{\text{5}\times \text{5}}(R))))$.
9. Calculate relative contrast: $C_{r}=\text{clip}((R-R_{b})/(R_{b}+\varepsilon ),-\text{1},\text{1})$.
10. Compute Sobel gradient response: $G_{x}=K_{x}*R,\;G_{y}=K_{y}*R,\;G=G_{x}+G_{y}$.
11. Generate modulation map: $A=\sigma (f_{\text{1}\times \text{1}}(\delta (\text{BN}(f_{\text{3}\times \text{3}}([X_{t},C_{r},G])))))$
12. Generate TCE output: $X_{\text{th}}=X_{t}⊙A$.
13. Generate Query, Key, and Value: $Q=P_{q}(X_{\text{th}}),=P_{kv}(X_{t})$.
14. Partition features into local windows: $\left\{X_{w}\},\{X_{\text{th},w}\}=\text{WindowPartition}(X_{t},X_{\text{th}}\right)$.
15. Compute local cross-feature attention: $A_{w}^{\left(j\right)}=\text{Dropout}(\text{Softmax}(Q_{w}^{\left(j\right)}{K_{w}^{\left(j\right)}}^{T}/d)).$
16. Reconstruct TGCA feature: $O=\text{BN}(\text{WindowReverse}(\{P_{o}({\text{Concat}}_{j=\text{1}}^{N_{h}}(A_{w}^{\left(j\right)}V_{w}^{\left(j\right)}))\})).$
17. Apply TGCA residual output: $\left(G_{t}=X_{t}+O\right)$.
18. Fuse enhanced branch: ${\hat{Y}}_{n+\text{1}}=Y_{n+\text{1}}+\sigma (\alpha )G_{t}$.
19. Generate TAF-C2f output: $X=F_{\text{out}}(F_{\text{3}\times \text{3}}(\text{Concat}(Y_{\text{2}},\ldots ,{\hat{Y}}_{n+\text{1}}))).$
20. **end for**
21. Aggregate multi-scale features through the neck network.
22. Predict class scores and bounding boxes using the detection head.
22. Apply non-maximum suppression to obtain final detection results D.
23. Return D.

**Fig 3:**
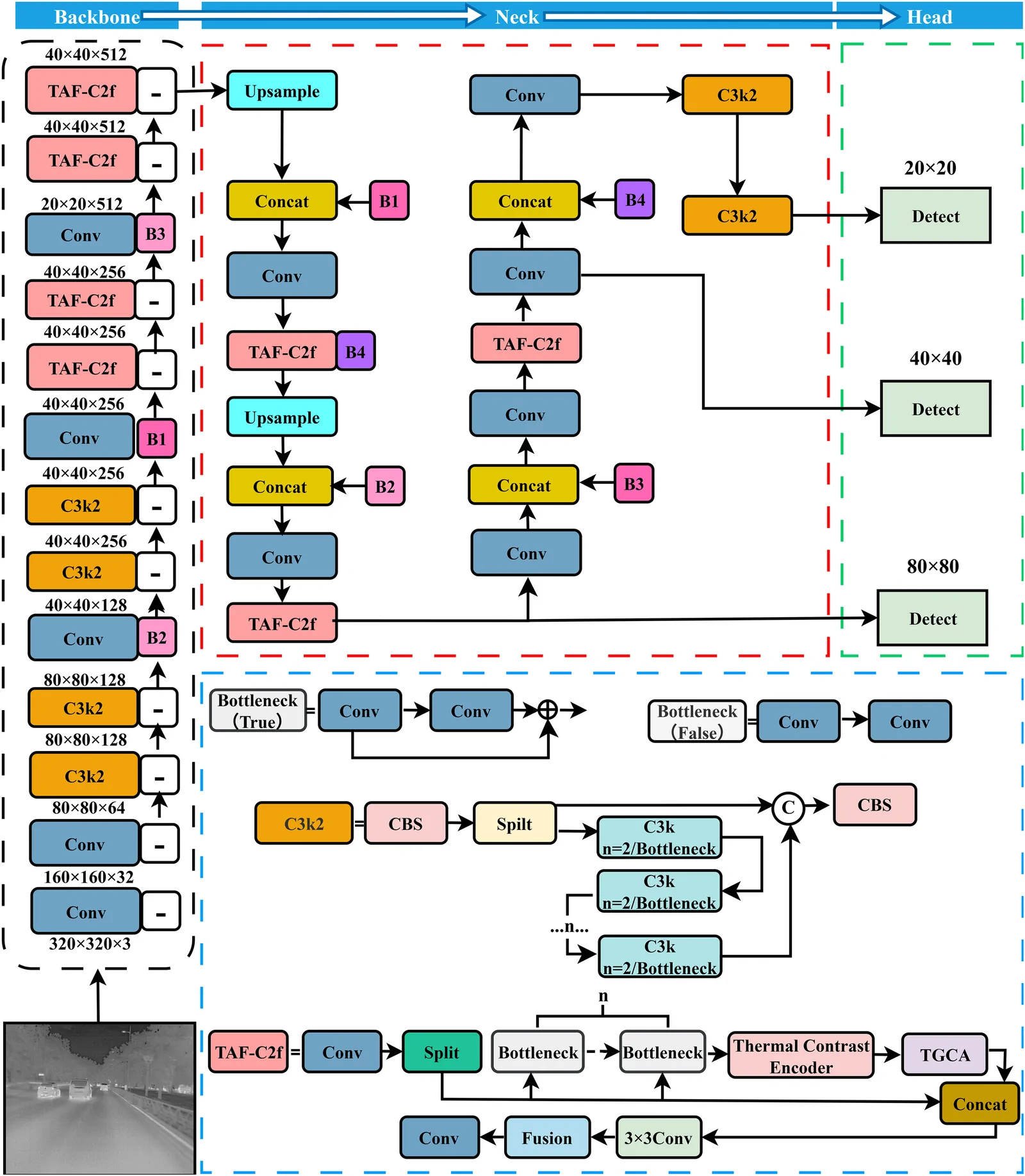
The structure of TCLNet.

TCLNet follows a typical one-stage detection architecture consisting of a backbone, neck, and detection head. The main modification is introduced in the backbone, where selected feature extraction blocks are replaced with the proposed TAF-C2f modules. By integrating the Thermal Contrast Encoder (TCE) and Thermal-Guided Cross-Feature Attention (TGCA), TAF-C2f enhances contrast-sensitive target responses and promotes local interactions between contrast-enhanced and semantic features.

The neck retains the original multi-scale feature aggregation structure of the baseline detector. Features from different backbone stages are fused through upsampling, concatenation, and convolutional operations. Since infrared-specific feature enhancement is mainly performed in the backbone, no additional complex attention modules are introduced into the neck, thereby limiting computational overhead.

The detection head also follows the baseline design and performs classification and bounding-box regression on multi-scale feature maps. Overall, TCLNet concentrates infrared-specific enhancement in the backbone while retaining lightweight feature fusion and prediction structures, improving the representation of weak infrared targets and suppressing background interference with limited additional computational cost.

## 4. Research methods

### 4.1. Dataset description

To evaluate TCLNet under different infrared detection conditions, experiments were conducted independently on M3FD, FLIR ADAS v2, and NUDT-SIRST1. M3FD and FLIR ADAS v2 were used for multi-class infrared object detection, whereas NUDT-SIRST1 provided an additional evaluation under weak and small-target conditions. A separate model was trained and evaluated on each dataset. Therefore, the NUDT-SIRST1 experiment is described as an independent small-target evaluation rather than a cross-dataset generalization experiment.

M3FD [38] contains 4,200 spatially aligned visible-thermal image pairs, corresponding to 8,400 individual images. Only the 4,200 thermal images were used in this study. The converted annotations contained 34,408 instances from six categories: person, car, bus, motorcycle, lamp, and truck.

FLIR ADAS v2 was collected from urban and suburban driving environments. The converted version used in this study, denoted as FLIR_2, contained 10 object categories and included substantial variations in target scale, thermal contrast, illumination, and background complexity.

NUDT-SIRST1 is a single-frame infrared small-target dataset containing weak targets against sky, ground, sea, and other complex backgrounds. The processed version used in this study contained 1,327 images and 1,863 annotated target instances.

New dataset partitions were created using an approximate training-validation-test ratio of 7:2:1. These were not the official dataset partitions. The partitions were fixed and shared by all compared methods. Table 2 reports the exact number of images and annotated instances in each subset. The corresponding image lists will be released to ensure reproducibility.

**Table 2:** Dataset partitioning used in the experiments.

Dataset	Classes	Training images	Validation images	Test images
M3FD	6	2,940	840	420
FLIR_2	10	8,321	2,377	1,188
NUDT-SIRST1	1	930	265	132

### 4.2. Evaluation metrics

To comprehensively evaluate detection performance and computational efficiency, precision (P), recall (R), average precision (AP), mean average precision (mAP), F1 score, miss rate (MR), model parameters, GFLOPs, and frames per second (FPS) were adopted as evaluation metrics. These metrics jointly characterize detection accuracy, target coverage, localization quality, computational complexity, and inference efficiency.

1) Precision and Recall: Precision is used to represent the proportion of the positive samples predicted by the detector that are actually positive, thereby measuring the accuracy of the model’s judgment in the detection of positive targets; Recall refers to the proportion of all actual positive samples that are successfully recognized as positive by the model, and is mainly used to evaluate the detector’s ability to capture targets. The specific definitions are as follows:

$$\begin{array}{c} \text{Precision }=\;\frac{\text{TP}}{\text{TP}+\text{FP}} \end{array}$$ (20)

$$\begin{array}{c} \text{Recall }=\;\frac{\text{TP}}{\text{TP}+\text{FN}} \end{array}$$ (21)

2) Average Precision (AP): As the core metric for evaluating the performance of models in the target detection task, average precision is used to measure the balance between precision and recall rates when the model detects specific types of targets. The value of AP is obtained by calculating the area under the precision-recall (PR) curve of this category (AUC), and its definition formula is as follows:

$$\begin{array}{c} \text{AP }=\;\int_{\text{0}}^{\text{1}}\text{P}(\text{r})\text{dr} \end{array}$$ (22)

3) Mean Average Precision (mAP): For a dataset containing C object categories, mean Average Precision is obtained by averaging the AP values over all categories. When the IoU threshold is fixed at 0.50, the metric is denoted as mAP50:

$$\begin{array}{c} \text{mAP}\;=\;\frac{\text{1}}{\text{C}}\sum_{\text{i}=\text{1}}^{\text{C}}{AP}_{\text{5}\text{0}}^{\left(c\right)} \end{array}$$ (23)

In object detection literature, $mAP_{\text{5}\text{0}}$ is also commonly written as mAP@0.5. These two notations refer to the same metric, namely the mean AP over all object categories at an IoU threshold of 0.50. To avoid ambiguity, $mAP_{\text{5}\text{0}}$ is used consistently throughout this paper.

Similarly, when the IoU threshold is fixed at 0.75, the corresponding metric is denoted as $mAP_{\text{7}\text{5}}$, which imposes a stricter requirement on bounding-box localization accuracy


$$\begin{array}{c} {maP}_{\text{7}\text{5}}=\frac{\text{1}}{C}\sum_{c=\text{1}}^{C}{AP}_{\text{7}\text{5}}^{\left(c\right)} \end{array}$$ (24)

To provide a more stringent and comprehensive evaluation of detection and localization performance, $mAP_{\text{5}\text{0}-\text{9}\text{5}}$ is calculated by averaging the mAP values over ten IoU thresholds ranging from 0.50 to 0.95 with an interval of 0.05:


$$\begin{array}{c} {maP}_{\text{5}\text{0}-\text{9}\text{5}}=\frac{\text{1}}{\text{1}\text{0}}\sum_{\tau \in \{\text{0}.\text{5}\text{0},\;\text{0}.\text{5}\text{5},...,\text{0}.\text{9}\text{5}\}}{maP}_{\tau } \end{array}$$ (25)

Therefore, mAP50 mainly reflects detection performance under an IoU threshold of 0.50, whereas mAP50 − 95 evaluates detection performance under a broader and stricter range of localization criteria.

4) F1 Score: The F1 score comprehensively considers precision and recall, and is the harmonic meaning of the two, allowing for a comprehensive assessment of the model’s detection performance. Its calculation formula is as follows:

$$\begin{array}{c} \text{F1 score }=\;\text{2}\times \frac{\text{Precision }\times \text{ Recall}}{\text{Precision }+\text{ Re}\text{call}} \end{array}$$ (26)

5) FPS: FPS (Frames Per Second, frames per second) is a key metric for measuring the speed of model inference, representing the number of images that the detector can process per second. The higher the FPS value, the stronger the real-time detection capability of the model. The calculation formula is as follows:

$$\begin{array}{c} \text{FPS }=\;\frac{\text{1}}{{\text{T}}_{\text{m}}} \end{array}$$ (27)

6) Miss Rate:Miss Rate (MR) measures the proportion of ground-truth targets that are not successfully detected under a specified operating threshold. It is defined as

$$\begin{array}{c} \text{MR}\;=\;\frac{\text{FN}}{\text{T}\text{P}+\text{FN}}=\text{1}-\text{R} \end{array}$$ (28)

A lower MR indicates better target coverage and fewer missed detections.

### 4.3. Training strategies and implementation details

All experiments were conducted using an NVIDIA GeForce RTX 4060 Ti GPU with 16 GB of memory. The software environment consisted of Python 3.10.18, PyTorch 2.7.1, CUDA 11.8, cuDNN 9.1.0, and Ultralytics 8.3.179. All images were resized to 320×320 pixels. The compared methods used the same dataset partitions, input resolution, and evaluation settings.

The models were trained from scratch for up to 200 epochs using stochastic gradient descent. The complete training configuration is summarized in Table 3. The random seed was fixed at 0, and deterministic training was enabled. Automatic mixed-precision training was used, whereas dataset caching, multi-scale training, checkpoint resumption, and pretrained initialization were disabled.

**Table 3:** Main training and inference settings.

Setting	Value
Hardware	NVIDIA GeForce RTX 4060 Ti, 16 GB
Software	Python 3.10.18; PyTorch 2.7.1; CUDA 11.8; Ultralytics 8.3.179
Input size	320 × 320
Training epochs	200
Batch size	8
Optimizer	SGD
Initial learning rate	0.01
Momentum	0.937
Weight decay	$\text{5}\times \text{1}{\text{0}}^{-\text{4}}$
Learning-rate schedule	Cosine decay
Initialization	Training from scratch
Main augmentation	Mosaic = 1.0; HSV = 0.015/0.7/0.4; translation = 0.1; scaling = 0.5; horizontal flipping = 0.5
Random seed	0
Deterministic training	True
Model selection	Highest validation fitness
FPS protocol	Batch size 1, FP32, 50 warm-ups, 5 repeated runs

## 5. Experiments and analysis

### 5.1. Results on M3FD and FLIR_2

To verify the effectiveness of the proposed TCLNet in the infrared target detection task, this paper conducted systematic experimental evaluations on two typical infrared datasets, M3FD and FLIR_2. The model performance was comprehensively analyzed using metrics such as precision (P), recall (R), mAP50, parameter quantity (Params), and inference speed (FPS), and compared with several mainstream YOLO series detection models. The relevant results are shown in Tables 4–10.

**Table 10:** Comprehensive detection performance comparison on M3FD and FLIR_2.

Method	M3FD				FLIR_2			
	mAP50	mAP75	mAP50–95	GFLOPs	mAP50	mAP75	mAP50–95	GFLOPs
YOLOv5n	47.7	34.92	33.30	1.768	40.1	20.45	21.55	1.770
YOLOv6n	42.6	33.40	31.90	2.945	33.2	17.74	18.08	2.946
YOLOv8n	48.3	37.14	35.20	2.023	38.3	21.62	22.82	2.025
YOLOv9t	49.4	37.02	35.29	1.901	40.8	20.23	21.89	1.904
YOLOv10n	45.7	34.08	33.21	1.633	34.6	20.65	21.54	1.633
YOLO11n	47.7	34.64	32.87	1.580	35.5	19.79	21.24	1.580
YOLO12n	47.7	34.28	32.19	1.581	34.1	19.75	21.27	1.584
YOLO13n	47	32.90	32.58	1.552	34.7	19.75	21.27	1.555
YOLO-SGF	41	20.73	21.80	1.176	28.9	11.94	14.48	1.179
YOLO-MIF	48.5	36.33	34.35	2.023	42.8	20.26	21.54	2.025
TCLNet	60.5	38.79	37.86	4.855	45.4	23.91	25.04	4.860

**Table 4:** Performance comparison between TCLNet and YOLOV8 series.

Method	M3FD					FLIR_2				
	P↑	R↑	mAP50↑	Params↓	Fps↑	P↑	R↑	mAP50↑	Params↓	Fps↑
YOLOv8n	0.613	0.445	48.3	2.33	303	0.548	0.368	38.3	3	210
YOLOv8s	0.711	0.494	53.5	11.12	175.4	0.624	0.39	44.3	11.13	147.05
YOLOv8m	0.719	0.543	56.8	25.84	84	0.685	0.458	51.3	25.84	64.51
YOLOv8l	0.702	0.595	61.1	43.61	58.5	0.68	0.487	53	43.61	44.24
TCLNet	0.81	0.532	60.5	2	156.25	0.608	0.416	45.4	2	211.76

**Table 5:** Performance comparison between TCLNet and YOLOV10 series.

Method	M3FD					FLIR_2				
	P↑	R↑	mAP50↑	Params↓	Fps↑	P↑	R↑	mAP50↑	Params↓	Fps↑
YOLOv10n	0.609	0.407	45.7	2.26	384.6	0.502	0.323	34.6	2.26	322.58
YOLOv10s	0.63	0.487	52.3	7.22	192.3	0.605	0.397	43.6	7.22	181.81
YOLO10m	0.715	0.532	58.5	15.31	86.2	0.624	0.434	44.9	15.32	65.78
YOLOv10l	0.747	0.509	56.3	24.31	55.9	0.583	0.458	45.2	24.32	48.07
TCLNet	0.81	0.532	60.5	2	156.25	0.608	0.416	45.4	2	211.76

**Table 6:** Performance comparison between TCLNet and YOLO11 series.

Method	M3FD					FLIR_2				
	P↑	R↑	mAP50↑	Params↓	Fps↑	P↑	R↑	mAP50↑	Params↓	Fps↑
YOLO11n	0.588	0.501	47.7	2.58	238.1	0.49	0.342	35.5	2.58	222.22
YOLO11s	0.663	0.503	50.6	9.41	169.5	0.631	0.396	43.5	9.41	123.45
YOLO11m	0.752	0.525	58.1	20.03	88.5	0.676	0.45	50	20.04	59.17
YOLO11l	0.714	0.562	62	25.28	72.5	0.658	0.455	50	25.29	47.84
TCLNet	0.81	0.532	60.5	2	156.25	0.608	0.416	45.4	2	211.76

**Table 7:** Performance comparison between TCLNet and YOLO12 series.

Method	M3FD					FLIR_2				
	P↑	R↑	mAP50↑	Params↓	Fps↑	P↑	R↑	mAP50↑	Params↓	Fps↑
YOLO12n	0.604	0.469	47.7	2.55	208.3	0.463	0.341	34.1	2.55	129.87
YOLO12s	0.73	0.506	54	9.23	126.6	0.621	0.396	43.2	9.23	60.6
YOLO12m	0.755	0.522	59.8	20.1	67.6	0.663	0.435	48.3	20.11	51.02
YOLO12l	0.744	0.554	60.5	26.34	45.7	0.616	0.436	47.4	26.35	30.03
TCLNet	0.81	0.532	60.5	2	156.25	0.608	0.416	45.4	2	211.76

**Table 8:** Performance comparison between TCLNet and YOLO13 series.

Method	M3FD					FLIR_2				
	P↑	R↑	mAP50↑	Params↓	Fps↑	P↑	R↑	mAP50↑	Params↓	Fps↑
YOLO13n	0.676	0.44	47	2.44	172.4	0.529	0.318	34.7	2.45	117.64
YOLO13s	0.74	0.5	56.6	9	95.2	0.65	0.395	44.7	9	75.75
YOLO13l	0.754	0.54	60.6	27.51	29.4	0.667	0.408	46.1	27.52	41.08
TCLNet	0.81	0.532	60.5	2	156.25	0.608	0.416	45.4	2	211.76

**Table 9:** Performance comparison of each method on the M3FD and FLIR_2 datasets.

Method	M3FD					FLIR_2				
	P↑	R↑	mAP50↑	Params↓	Fps↑	P↑	R↑	mAP50↑	Params↓	Fps↑
YOLOv5n	0.655	0.437	47.7	2.5	277.77	0.62	0.365	40.1	2.5	384.61
YOLOv6n	0.555	0.417	42.6	4.23	285.71	0.538	0.318	33.2	4.23	333.33
YOLOv8n	0.613	0.445	48.3	2.33	303	0.548	0.368	38.3	3	210
YOLOv9t	0.646	0.476	49.4	1.97	263.15	0.639	0.374	40.8	1.97	312.5
YOLOv10n	0.609	0.407	45.7	2.26	384.6	0.502	0.323	34.6	2.26	322.58
YOLO11n	0.588	0.501	47.7	2.58	238.1	0.49	0.342	35.5	2.58	222.22
YOLO12n	0.604	0.469	47.7	2.55	208.3	0.463	0.341	34.1	2.55	129.87
YOLO13n	0.676	0.44	47	2.44	172.4	0.529	0.318	34.7	2.45	117.64
YOLO-SGF	0.575	0.405	41	1.48	303	0.451	0.291	28.9	1.48	303
YOLO-MIF	0.636	0.472	48.5	3	208.33	0.573	0.407	42.8	3	208.33
TCLNet	0.81	0.532	60.5	2	156.25	0.608	0.416	45.4	2	211.76

On the M3FD dataset, the mAP50 of TCLNet reached 60.5%, which was 12.8% higher than the lightweight baseline models YOLO11n and YOLO12n. Meanwhile, with only approximately 2M of parameter quantity, the model still maintained a high detection accuracy, demonstrating good feature expression ability and lightweight advantages. Compared with YOLO13n, in the case of a parameter reduction of approximately 18.0%, the mAP50 still increased by 13.5%, further indicating that the proposed method has better detection performance in a small model scale. On the FLIR_2 dataset, TCLNet also showed stable performance, with an mAP50 of 45.4%, which was 10.8% and 4.6% higher than YOLOv10n and YOLO11n respectively, indicating that this method has certain generalization ability in cross-scenario infrared data.

From the perspective of model complexity and inference efficiency, the parameter quantity of TCLNet is significantly lower than medium-sized models such as YOLOv8s and YOLOv10s, being only about 21.3% of YOLO11s, but still has competitive detection accuracy. In terms of inference speed, TCLNet achieved inference speeds of 156.25 FPS on the M3FD dataset and 211.76 FPS on the FLIR_2 dataset, which can meet real-time detection requirements. The above results indicate that the proposed method effectively controls the computational cost while ensuring detection performance, achieving a good balance between accuracy and efficiency.

Furthermore, to verify the advanced nature of the method, this paper compares TCLNet with several mainstream lightweight detection models, including YOLOv5n, YOLOv6n, YOLOv8n, YOLOv9t, YOLOv10n, YOLO11n, YOLO12n, YOLO13n [39], as well as the improved models YOLO-MIF [40] and YOLO-SGF [41,42], etc. As shown in Table 9, on the M3FD dataset, compared with the superior YOLOv9t and YOLO-MIF, TCLNet achieves an improvement of 11.1% and 12.0% respectively; on the FLIR_2 dataset, the mAP50 of TCLNet reaches 45.4%, which is an increase of 2.6% compared to YOLO-MIF. Meanwhile, while maintaining approximately 2M parameters, TCLNet still has a significant advantage in detection accuracy, demonstrating strong comprehensive performance.Meanwhile, the results in Table 10 also show that TCLNet maintains approximately 2M parameters while achieving superior detection accuracy, demonstrating a favorable trade-off between detection performance and model complexity.

In summary, TCLNet has demonstrated stable and superior detection performance on multiple infrared datasets. While maintaining a low model complexity and high inference speed, it effectively enhances the target detection capability in low signal-to-noise ratio and complex background scenarios, thereby verifying the effectiveness of the proposed thermal physical feature modeling and thermal-guided attention mechanism.

### 5.2. Results on NUDT-SIRST1

To further evaluate the performance of TCLNet under weak and small-target conditions, experiments were conducted on the NUDT-SIRST1 dataset. This dataset is characterized by low signal-to-clutter ratios, small target sizes, and complex backgrounds, presenting substantial challenges for target representation and localization. Each model was trained and evaluated independently on NUDT-SIRST1 using the same dataset partitions. The results are presented in Table 11 and Table 12.

**Table 11:** Performance comparison of different methods on NUDT-SIRST1.

Method	P↑	R↑	mAP50↑	Params↓	Fps↑
YOLO12n	0.835	0.726	83.9	2.55	149.25
YOLO-SGF	0.761	0.803	81.5	1.48	322.58
YOLO-MIF	0.8087	0.6233	71.82	3	196.07
TCLNet	0.835	0.798	91.4	2	72.64

**Table 12:** AP50, AP75, mAP50–95, and APsmall comparison on NUDT-SIRST1.

Method	AP50	AP75	mAP50-95	mAPsmall	GFLOPs
YOLO12n	83.9	37.85	40.65	40.65	1.580
YOLO-SGF	81.5	27.37	36.19	36.19	1.175
YOLO-MIF	71.82	34.09	38.04	38.04	2.021
TCLNet	91.4	49.15	50.52	50.52	4.852

TCLNet achieved the highest mAP50 of 91.4%, exceeding YOLO12n, the strongest baseline in terms of mAP50, by 7.5 percentage points. It also achieved a precision of 0.835, tying with YOLO12n for the highest precision among the evaluated methods and outperforming YOLO-SGF and YOLO-MIF by 0.074 and 0.0263, respectively.

For recall, TCLNet achieved 0.798, which was 0.072 higher than YOLO12n and 0.1747 higher than YOLO-MIF. Although its recall was 0.005 lower than that of YOLO-SGF, TCLNet produced a substantially higher mAP50, exceeding YOLO-SGF by 9.9 percentage points. These results indicate that TCLNet achieved a more favorable balance between detection precision, target coverage, and overall detection accuracy.

The improved performance is consistent with the intended roles of the thermal-contrast feature extraction and thermal-guided attention mechanisms. These components enhance the representation of weak target regions while reducing interference from complex infrared backgrounds. However, the present results demonstrate an association between the proposed design and improved detection performance rather than independently establishing the contribution of each component; their individual effects are examined through the ablation experiments in Section 5.3.

TCLNet contained approximately 2.00 million parameters, representing a reduction of 21.6% relative to YOLO12n and 33.3% relative to YOLO-MIF. Its parameter count was higher than that of YOLO-SGF, which contained 1.48 million parameters. TCLNet achieved a reported inference speed of 72.64 FPS, lower than the other evaluated models. This result indicates that the proposed feature-enhancement and attention operations introduce additional computational latency. Nevertheless, TCLNet provided the highest mAP50 while retaining a compact parameter count, demonstrating a trade-off that favors detection accuracy under challenging infrared small-target conditions.

Overall, TCLNet achieved the highest mAP50 and joint-highest precision among the converged models evaluated on NUDT-SIRST1. Although it did not achieve the highest recall, smallest model size, or fastest inference speed, it provided the strongest overall detection accuracy, supporting its effectiveness for weak infrared small-target detection.

### 5.3. Ablation analysis

To evaluate the effectiveness of the proposed Thermal Contrast Encoder (TCE) and Thermal-Guided Cross-Feature Attention (TGCA), ablation experiments were conducted on the M3FD dataset using the corresponding lightweight baseline model. TCE and TGCA were introduced individually and jointly to investigate their respective contributions and complementary effects. The experimental results are summarized in Table 13.

**Table 13:** Performance Comparison of baseline combinations under Different Strategies.

Method	TCE	TGCA	p	R	mAP50	Params	Fps
Baseline	–	–	0.724	0.472	51	1.743	232.5
	**√**	–	0.804	0.436	53.5	1.811	277.7
	–	√	0.794	0.452	52.2	1.936	169.4
	√	√	0.81	0.532	60.5	2.007	156.25

As shown in Table 13, the baseline model achieves a precision of 0.724, a recall of 0.472, and an mAP50 of 51.0%, with 1.743M parameters and an inference speed of 232.5 FPS. After introducing TCE alone, the precision increases from 0.724 to 0.804, while the mAP50 improves from 51.0% to 53.5%, corresponding to a gain of 2.5 percentage points. Meanwhile, the recall decreases slightly from 0.472 to 0.436. This result suggests that TCE mainly strengthens the discriminative representation of thermal targets and suppresses background interference, thereby improving prediction precision, although its contribution to target coverage is relatively limited when used independently. The introduction of TCE increases the parameter count only slightly, from 1.743M to 1.811M. The corresponding model achieves an inference speed of 277.7 FPS.

When only TGCA is introduced, the model achieves a precision of 0.794, a recall of 0.452, and an mAP50 of 52.2%. Compared with the baseline, the precision increases by 0.070 and the mAP50 improves by 1.2 percentage points, whereas the recall decreases slightly by 0.020. These results indicate that TGCA facilitates interaction between contrast-sensitive features and high-level semantic representations, which improves feature discrimination to a certain extent. However, TGCA alone provides only a moderate improvement in overall detection performance. The model contains 1.936M parameters and achieves an inference speed of 169.4 FPS.

When TCE and TGCA are jointly incorporated, the model achieves the best overall detection performance, with a precision of 0.810, a recall of 0.532, and an mAP50 of 60.5%. Compared with the baseline, the precision and recall increase by 0.086 and 0.060, respectively, while the mAP50 improves by 9.5 percentage points. Notably, the improvement obtained by jointly introducing TCE and TGCA is substantially greater than that obtained by either module individually. This indicates a clear complementary relationship between the two modules. Specifically, TCE enhances contrast-sensitive thermal target representations, while TGCA further promotes the interaction between the enhanced features and semantic features. Their joint operation enables the network to preserve more useful target information while suppressing background interference, thereby improving both feature discrimination and target localization.

In terms of model complexity, the complete model contains approximately 2.007M parameters, representing an increase of only 0.257M compared with the 1.743M-parameter baseline. Despite the additional feature-enhancement and attention operations, the overall parameter scale remains lightweight. The complete model achieves an inference speed of 156.25 FPS, which is lower than that of the baseline and the individual-module variants but remains sufficient for high-speed infrared object detection. Considering the substantial improvement in detection accuracy, particularly the increase in mAP50 from 51.0% to 60.5%, the additional computational cost is acceptable.

Overall, the ablation results demonstrate that both TCE and TGCA contribute positively to the detection performance of the proposed TCLNet. TCE mainly enhances thermal contrast-sensitive representations, whereas TGCA improves feature interaction and semantic guidance. More importantly, their joint integration produces a clear complementary effect, simultaneously improving precision and recall and increasing the mAP50 by 9.5 percentage points over the baseline. These results verify the effectiveness of combining thermal contrast enhancement with cross-feature attention for infrared object detection.

### 5.4. Visual and per-class analysis

To further examine the detection behavior of TCLNet under different infrared scenarios, qualitative comparisons were conducted with representative lightweight detectors, on M3FD, FLIR_2, and NUDT-SIRST1. Representative visualization results are shown in Figs 4–6, while Tables 14–16 report the corresponding per-class detection performance of TCLNet.

**Table 14:** Per-class detection performance of TCLNet on M3FD.

Class	Instances	AP50	AP75	mAP50-95
People	1,197	64.86	33.06	34.49
Car	1,799	79.23	56.87	52.57
Bus	62	77.52	66.34	60.65
Motorcycle	188	28.67	7.13	11.21
Lamp	48	40.44	22.63	23.11
Truck	101	66.56	46.70	45.15

**Table 15:** Per-class detection performance of TCLNet on FLIR_2.

Class	Instances	AP50	AP75	mAP50–95
person	11,077	61.35	28.26	30.86
bike	1,556	49.28	22.34	25.17
car	16,232	68.92	44.65	43.91
motor	256	50.43	21.08	25.21
bus	486	69.36	53.71	45.83
train	128	45.36	27.62	27.84
truck	164	27.12	19.84	18.63
light	3,595	38.54	13.27	17.74
hydrant	253	17.68	2.86	7.15
sign	4,726	25.96	13.11	13.62

**Table 16:** Per-class detection performance of TCLNet on SIRST.

Class	Instances	mAP50	mAP75	mAP50–95
Target	186	91.4	49.15	50.52

**Fig 4:**
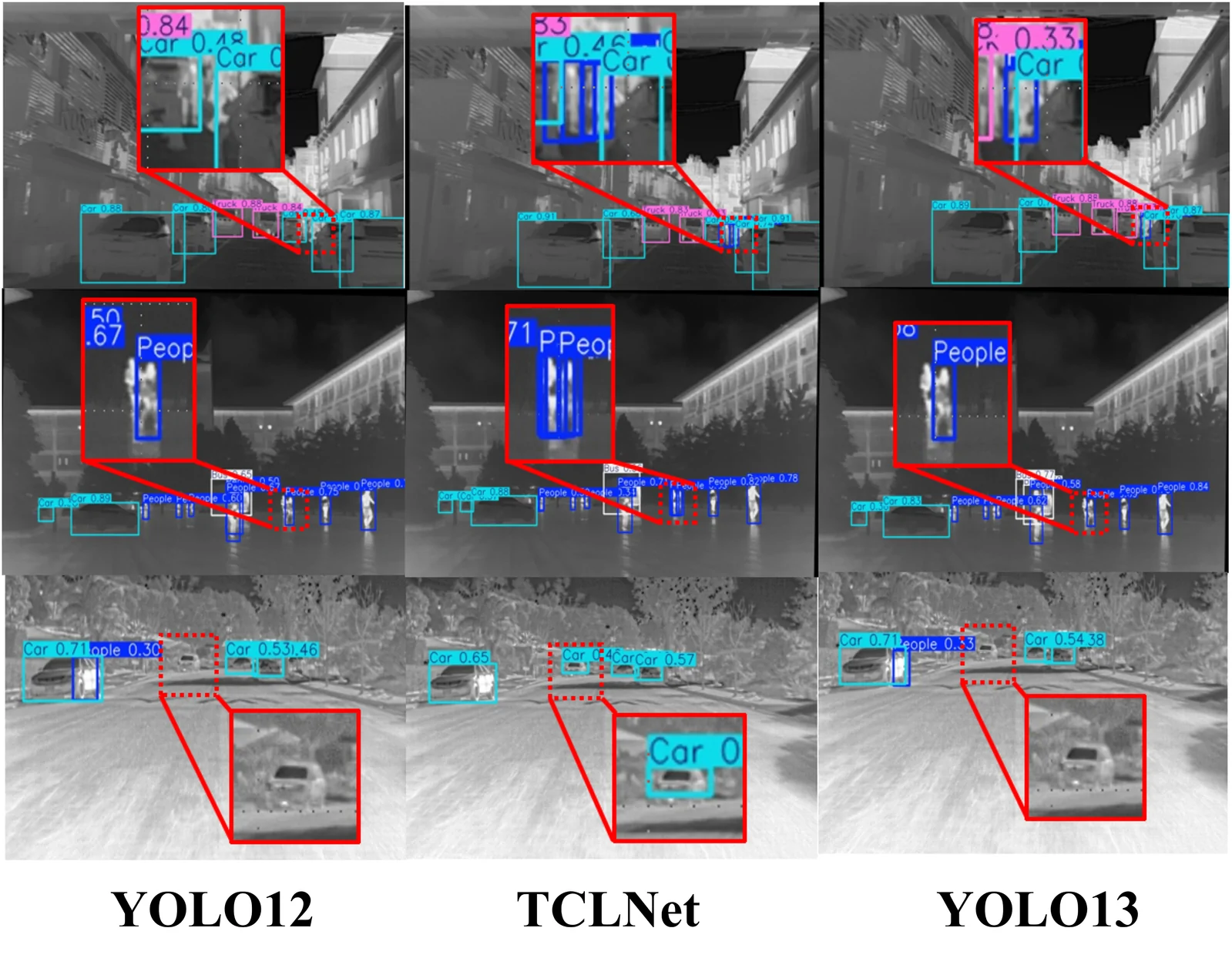
Visualization results of different models on the M3FD dataset.

**Fig 5:**
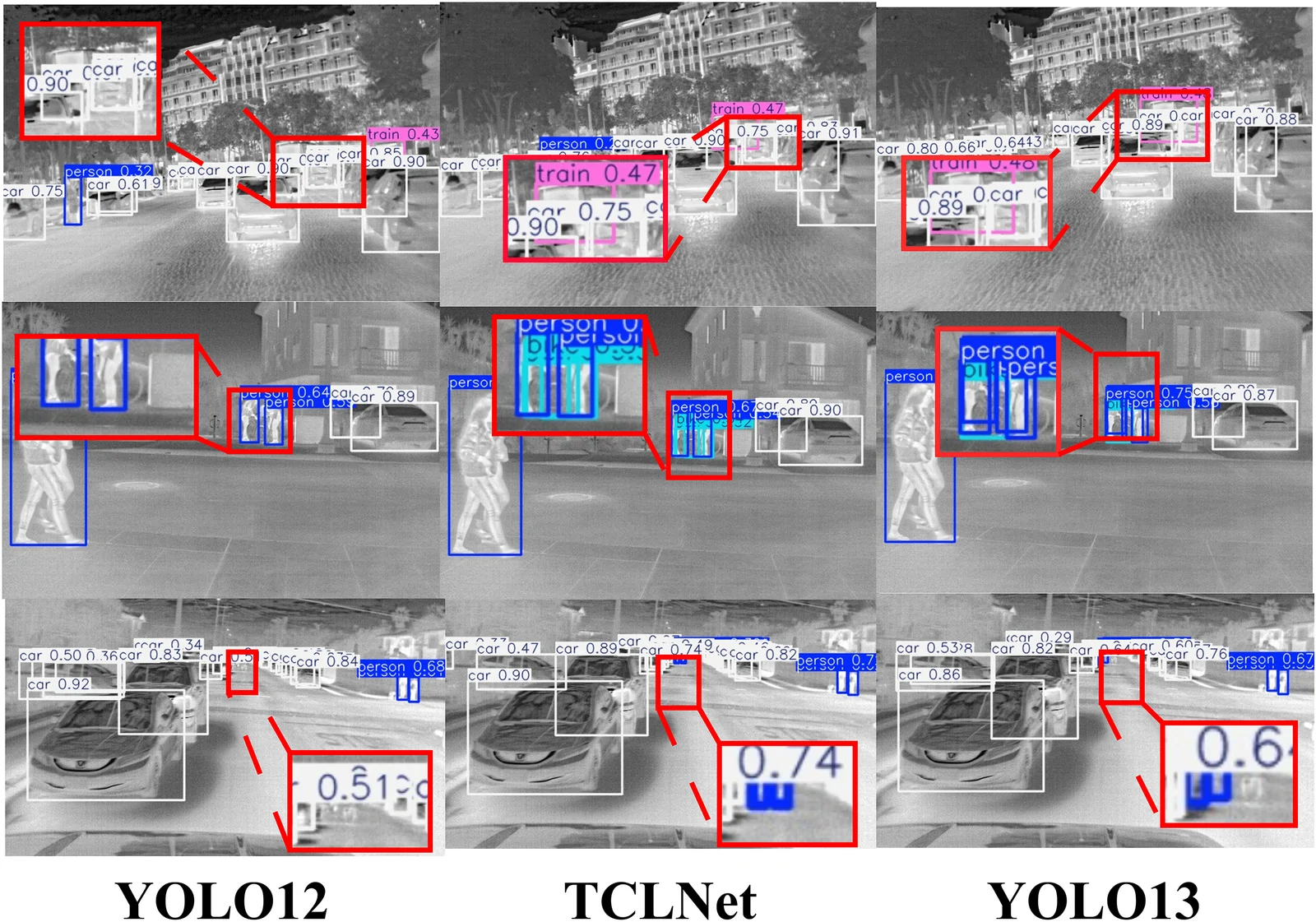
Visual results of different models on the FLIR_2 dataset.

**Fig 6:**
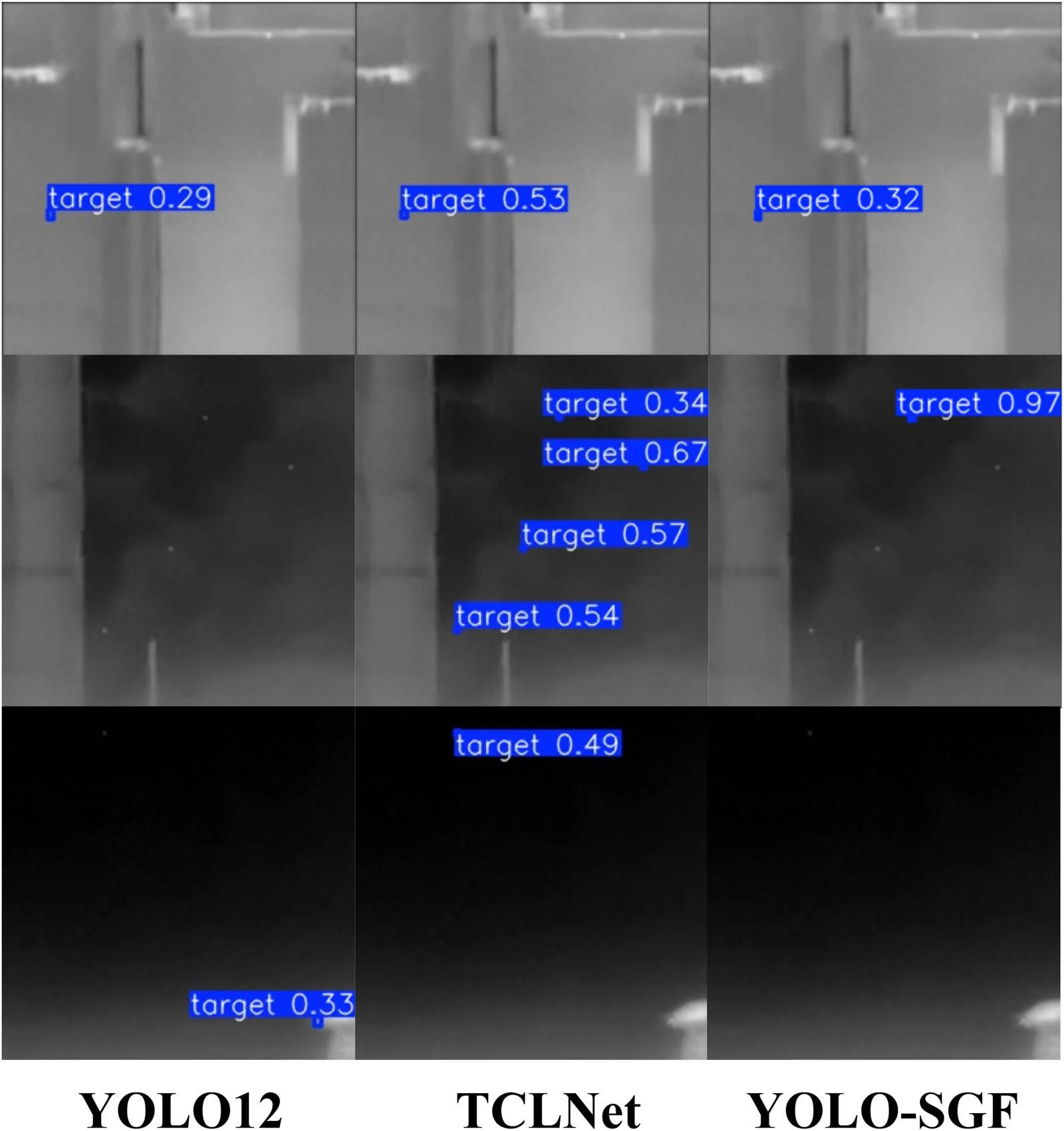
Visual results of different models on the SIRST dataset.

On the M3FD dataset, as shown in Fig 4, TCLNet provides relatively complete detections in urban scenes containing pedestrians, vehicles, and multiple objects. In particular, the model maintains stable responses for low-contrast and relatively small targets while reducing missed detections and inaccurate localization compared with the selected baseline methods. This qualitative observation is consistent with the intended role of TCE, which enhances local target-background contrast-sensitive features and improves the representation of weak infrared targets.

The per-class results in Table 14 further show that TCLNet performs strongly on major categories such as Car, Bus, People, and Truck. Car and Bus achieve AP50 values of 79.23% and 77.52%, respectively. In contrast, Motorcycle and Lamp remain more difficult, with AP50 values of 28.67% and 40.44%. The particularly low AP75 of Motorcycle indicates that accurate localization remains challenging for small-scale objects with limited spatial information.

On the FLIR_2 dataset, Fig 5 shows that TCLNet remains effective in scenes containing multiple object categories and cluttered backgrounds. The model produces relatively stable detections for pedestrians, vehicles, and other common targets, while some comparison models exhibit missed detections or incomplete localization in crowded regions. This behavior is consistent with the intended function of TGCA, which promotes local interaction between contrast-enhanced and semantic features and helps suppress irrelevant background responses.

As shown in Table 15, TCLNet achieves relatively high AP50 values for Bus, Car, and Person, reaching 69.36%, 68.92%, and 61.35%, respectively. In comparison, Hydrant, Sign, and Truck show lower detection performance. These results indicate that target category, scale, appearance variability, and background interference remain important factors affecting detection difficulty on FLIR_2.

On NUDT-SIRST1, TCLNet demonstrates relatively stable detection of weak and extremely small infrared targets, as illustrated in Fig 6. Compared with the selected baseline methods, TCLNet is able to retain more valid target responses in scenes where the targets occupy only a few pixels and exhibit weak contrast against the background. Nevertheless, some small targets remain difficult to localize accurately because their spatial structure and boundary information are highly limited.

The quantitative results in Table 16 are consistent with this observation. TCLNet achieves an AP50 of 91.4%, while the AP75 decreases to 49.15%. The substantial difference between AP50 and AP75 indicates that, although most small targets can be successfully detected under a relatively relaxed IoU threshold, precise bounding-box localization remains difficult for extremely small targets.

Overall, the visual and per-class analyses show that TCLNet maintains relatively stable detection performance across different infrared scenarios. However, motorcycles and other small-scale objects on M3FD, several difficult categories on FLIR_2, and high-IoU localization of extremely small targets on NUDT-SIRST1 remain challenging. These observations motivate the more detailed robustness and failure-case analysis presented in Section 5.5.

### 5.5. Failure-case analysis under challenging infrared conditions

To further evaluate the robustness of TCLNet under challenging infrared conditions, quantitative and qualitative analyses were conducted on M3FD, FLIR_2, and NUDT-SIRST1. The analysis focuses on small-object and dense-scene conditions. Following the COCO-style object-size criterion adopted in our evaluation, a target was considered a small object when its ground-truth bounding-box area was smaller than $\text{3}{\text{2}}^{\text{2}}$ pixels in the original image coordinate system. A dense scene was defined as an image containing at least 10 annotated object instances. For NUDT-SIRST1, the complete test split was evaluated within the same small-object area range. Miss Rate (MR) was used to quantify missed detections, with a lower value indicating better target coverage.

As shown in Table 17, the quantitative results indicate that small-object detection remains a major challenge on M3FD and FLIR_2. Compared with dense scenes, the small-object subsets exhibit lower mAP values and higher miss rates, indicating that weak spatial information and limited target size significantly affect both detection and localization accuracy. In contrast, the stronger performance on NUDT-SIRST1 suggests that target size alone does not determine detection difficulty; category diversity and background clutter also play important roles.

**Table 17:** Quantitative performance of TCLNet under challenging infrared conditions.

Dataset	Condition	images	Instances	mAP50	mAP75	mAP50–95	MR
M3FD	Small objects	260	1,384	17.60	3.82	6.50	67.99
M3FD	Dense scenes	148	2,303	55.02	32.32	33.03	36.65
FLIR_2	Small objects	1,099	14,111	23.53	8.75	10.86	64.20
FLIR_2	Dense scenes	837	17,369	32.16	17.54	17.87	50.66
NUDT-SIRST1	Small-target benchmark	132	186	93.08	49.48	50.77	10.75

Representative failure cases are shown in Fig 7. Distant and extremely small targets may suffer from missed detections or inaccurate localization because spatial details are weakened during feature downsampling. Low-contrast targets may produce low-confidence predictions due to insufficient target-background intensity differences, while cluttered backgrounds can generate target-like responses and cause false positives or redundant boxes.

**Fig 7:**
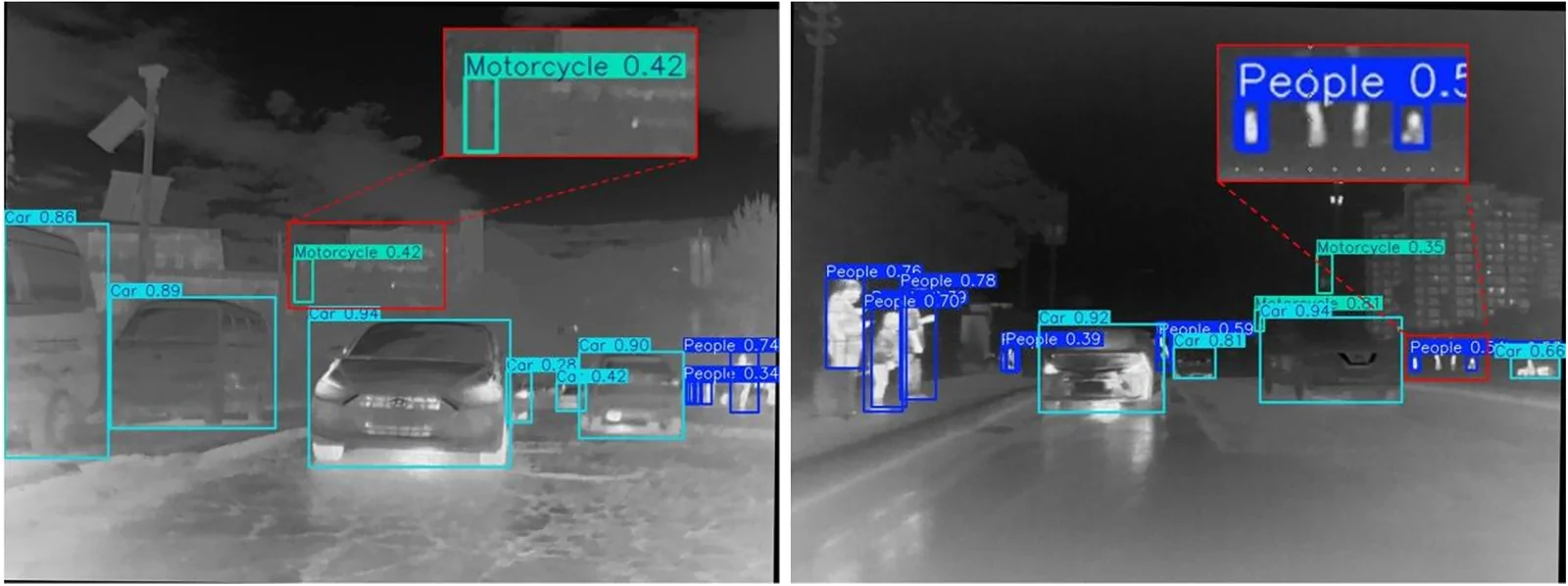
Identification Error Results.

Overall, the remaining errors are mainly related to spatial-detail loss, weak target-background contrast, ambiguous boundaries, and background interference. Future work will focus on higher-resolution feature preservation, adaptive small-target enhancement, hard-negative mining, and improved bounding-box regression and post-processing strategies.

## 6. Conclusion

This paper proposes TCLNet, a Thermal Contrast-Aware Lightweight Network for infrared object detection. Based on the lightweight YOLO12n framework, TCLNet integrates a Thermal Contrast Encoder (TCE) and a Thermal-Guided Cross-Feature Attention (TGCA) mechanism to enhance local contrast-sensitive target representations and promote interactions between contrast-enhanced and semantic features. The proposed design improves infrared target discrimination while maintaining a compact model size.

Experimental results on M3FD, FLIR_2, and NUDT-SIRST1 demonstrate the effectiveness of TCLNet under independently trained and evaluated benchmark settings. TCLNet achieves mAP50 values of 60.5% and 45.4% on M3FD and FLIR_2, respectively, while maintaining approximately 2M parameters. On NUDT-SIRST1, TCLNet also achieves strong detection performance for weak and small infrared targets. The ablation experiments further show that the joint use of TCE and TGCA provides complementary gains, increasing mAP50 from 51.0% to 60.5%. Although the additional feature-enhancement and local-attention operations increase computational cost, TCLNet achieves a favorable trade-off between detection accuracy and model complexity.

Further robustness and failure-case analyses show that extremely small targets, weak target-background contrast, ambiguous boundaries, and cluttered backgrounds remain challenging. Spatial-detail loss during feature downsampling may lead to missed detections and inaccurate localization, while target-like background responses can cause false positives and redundant detections. Future work will therefore focus on higher-resolution and scale-aware feature representations, adaptive contrast enhancement, hard-negative mining, and improved bounding-box regression and post-processing. Multimodal visible-infrared information will also be explored to further improve performance in complex practical environments.
